# Tubridge flow diverter for the treatment of small and medium aneurysms

**DOI:** 10.3389/fneur.2023.1054631

**Published:** 2023-01-30

**Authors:** Dajiang Xie, Heng Yang, Li Zhao, Xin Ye, Shuxu Yang, Chao Gao, Yanlong Tian, Wei Ni, Yuxiang Gu

**Affiliations:** ^1^Department of Neurosurgery, Huashan Hospital, Shanghai Medical College, Fudan University, Shanghai, China; ^2^Department of Neurosurgery, Sir Run Run Shaw Hospital, College of Medical Sciences, Zhejiang University, Hangzhou, China; ^3^Department of Gastroenterology, Hangzhou Red Cross Hospital/Integrated Traditional Chinese and Western Medicine Hospital of Zhejiang Province, Zhejiang Chinese Medical University, Hangzhou, China

**Keywords:** Tubridge, flow diverter, small aneurysm, medium aneurysm, tandem aneurysm

## Abstract

**Background:**

Tubridge flow diverter is a widely used device aimed at reconstructing parent arteries and occluding complex aneurysms in China. The experience of Tubridge in treating small and medium aneurysms is still limited. In this study, we aimed to evaluate the safety and efficacy of the Tubridge flow diverter for the treatment of the two types of aneurysms.

**Methods:**

We reviewed the clinical records of aneurysms treated with a Tubridge flow diverter between 2018 and 2021 in a national cerebrovascular disease center. Cases were divided into small and medium aneurysms according to aneurysm size. The therapeutic process, occlusion rate, and clinical outcome were compared.

**Results:**

In total, 57 patients and 77 aneurysms were identified. The patients were divided into two groups: small aneurysms (39 patients, 54 aneurysms) and medium aneurysms (18 patients, 23 aneurysms). There were 19 patients with tandem aneurysms (a total of 39 aneurysms) in the two groups, among which 15 patients (30 aneurysms) were in the small aneurysm group and four patients (nine aneurysms) were in the medium aneurysm group. The results show that the mean maximal diameter/neck in the small and medium aneurysms was 3.68/3.25 and 7.61/6.24 mm, respectively. In total, 57 Tubridge flow diverters were successfully implanted without unfolding failure, and there were six patients with new mild cerebral infarction in the small aneurysm group. The complete occlusion rate on the last angiographic follow-up was achieved in 88.46% of the small aneurysms group and 81.82% of the medium aneurysms group. The complete occlusion rate of patients with tandem aneurysms in the last angiographic follow-up was 86.67% (13/15) of the small aneurysms group and 50% (2/4) of the medium aneurysm group. Intracranial hemorrhage was nonencountered in the two groups.

**Conclusion:**

Our preliminary experience suggests that the Tubridge flow diverter might be a safe and effective treatment for small and medium aneurysms along the internal carotid artery. Long stents may increase the risk of cerebral infarction. Adequate evidence is required to clarify the definite indications and complications in a multicenter randomized controlled trial with a long-term follow-up.

## Introduction

Intracranial aneurysm is one of the most common cerebrovascular diseases. The main management methods include clipping, endovascular therapy, and close follow-up. At present, a blood flow diverter, a new effective means of endovascular therapy, is widely used in the treatment of large or giant aneurysms, but there are few reports on the treatment of small aneurysms and medium aneurysms (1). These aneurysms are most commonly found along the internal carotid artery.

Tubridge is a new type of flow diverter device developed by MicroPort Medical Company (Shanghai, China) based on previous hemodynamic studies of intracranial aneurysms that aims at treating complex aneurysms that are difficult to access by clipping or conventional endovascular treatment, such as large or giant aneurysms, and providing more treatment options for neurointerventionalists and neurosurgeons (2). Numerous studies have since demonstrated its safety and efficacy in the treatment of aneurysms with varying morphologies and anatomic locations.

Currently, there is no study evaluating endovascular treatment of small and medium aneurysms with a Tubridge flow diverter. Therefore, we collected the clinical records, compared the outcomes following Tubridge deployment in patients in the two groups, reviewed the current literature, and discussed its future use.

## Materials and methods

### Patient selection

The institutional review board of the hospital approved this study and waived the requirement for patient-informed consent due to its retrospective design. We collected the cases of unruptured intracranial aneurysms treated with Tubridge flow diverter from 2018 to 2021 except large (>10 mm) and giant (≥ 25 mm) aneurysms. Patients were then divided into two groups: small aneurysm (≤5 mm) and medium aneurysm (≤10 mm).

### Anticoagulation and antiplatelet management

Each patient received systemic heparin after the placement of the microcatheter. The activated clotting time was maintained at 2–3 times the baseline throughout the procedure. Each patient received dual antiplatelet drugs (100 mg/day aspirin plus 75 mg/day clopidogrel) for at least 3 days before the procedure. The intravenous loading dose of tirofiban was 5 μg/kg for 3–5 min and the maintenance dose of tirofiban was 0.1 μg/ (kg·min) for 24 h after Tubridge flow-diverter deployment. A postoperative antiplatelet regimen was administered as follows: ≤3 months, 100 mg/day aspirin + 75 mg/day clopidogrel; >3 months, 100 mg/day aspirin, and 1 year.

### Tubridge flow diverter and implantation/coiling procedure

The Tubridge flow diverter is a braided, self-expanding stent device with flared ends. Current Tubridge flow diverters are available in various diameters (2.5–6.5 mm) and lengths (12–45 mm). The Tubridge is composed of nickel–titanium and two platinum–iridium microfilaments. All Tubridge flow diverters were designed with a pore size of 0.040–0.050 mm^2^ to provide high metal coverage (~30.0%−35.0%) at the aneurysm neck after full opening.

The Tubridge is mounted to a delivery wire and constrained within a removable sheath. The tip of the delivery wire is J-shaped, which is designed to help prevent vascular injury, facilitate microcatheter removal through previous devices, and deploy a second flow diverter.

A Tubridge flow-diverter device was introduced *via* the 0.029 inch diameter microcatheter into the target zone. The device began to expand in the artery and was deployed by pushing the delivery wire and simultaneously drawing the microcatheter. In general, the shortening rate after complete deployment is approximately < 50.0%, depending on the size of the Tubridge and any discrepancies between the proximal and distal vessel diameters. The device can be retracted until released to the marker in the middle of Tubridge. Before the stent is completely released, coiling embolization is feasible. After the first flow diverter was deployed, a second diverter was considered if necessary.

### Angiographic evaluation and clinical outcome

An angiographic evaluation was assessed with digital subtraction angiography based on protocols. The angiographic results obtained immediately after the procedures were based on retention or decreased filling of the contrast agent in the aneurysms. Aneurysm occlusion on follow-up angiography imaging was assessed by the treating interventionalist. The occlusion rate was categorized as complete occlusion (100%), near-complete occlusion (90%−100%), and partial occlusion (< 90%). Clinical outcomes were assessed by using the modified Rankin Scale at the last follow-up.

### Statistical analysis

Continuous variables, including demographics, aneurysm characteristics, and procedural characteristics, were represented as mean ± SD. Categorical data were presented as numbers and percentages. Continuous variables were compared using the unpaired Student's *t*-test or the Mann–Whitney test and categorical variables were compared using the Fisher's exact test or, if there were more than two possible categories, using the chi-squared test. Statistical significance was defined as *p* < 0.05.

## Results

### Patient characteristics

In total, 57 patients (38 women and 19 men) and 77 aneurysms with a mean age of 56 years (ranging from 33 to 81 years) and 40.35% of high BMI (≥24) were identified from 2018 to 2021. The case numbers were 39 and 18 in the small and medium aneurysm groups, respectively. The pretreatment mRS score of 41.03% of patients in the small aneurysm group and 44.44% of patients in the medium aneurysm group was 1. The proportion of BMI ≥24 in the medium aneurysm group was significantly higher than that in the small aneurysm group (*p* < 0.05). There was no significant difference in age, gender, or pretreatment mRS between the two groups (Table 1).

**Table 1:** Baseline characteristics of enrolled patients.

**Parameters**	**Small aneurysm**	**Medium aneurysm**	***p*-Value**
Cases	39 (68.42%)	18 (31.58%)	
Age (years)	55 ± 9	56 ± 10	0.92
Gender (M/F)	12/27	7/11	0.55
BMI ≥24	12 (30.77%)	11 (61.11%)	< 0.05^*^
No. of aneurysms	54	23	0.83
**Side of aneurysms**			
Right	29 (53.70%)	11 (47.83%)	0.64
Left	25 (46.30%)	12 (52.17%)	
**Aneurysm locations**			
Middle cerebral artery	1 (2.56%)	0 (0%)	0.49
Internal carotid artery	38 (97.44%)	18 (100%)	
**Aneurysm shape**			
Saccular	53 (98.15%)	23 (100%)	0.51
Fusiform	1 (1.85%)	0 (0%)	
Dissecting	0 (0%)	0 (0%)	
**Aneurysm measurements**			
Mean maximal diameter	3.68 ± 0.82	7.61 ± 1.64	< 0.05^*^
Neck size	3.25 ± 0.96	6.24 ± 1.97	
**Parent artery diameter**			
Distal diameter	3.55 ± 0.45	3.56 ± 0.40	0.99
Proximal diameter	4.21 ± 0.49	4.47 ± 0.46	0.93

* Statistically significant.

### Aneurysm characteristics

The aneurysm numbers were 54 and 23 in the small and medium aneurysm groups, respectively. There were 29 and 11 cases on the right side in the small and medium aneurysm groups, respectively (*p* = 0.64). Aneurysms were primarily saccular and located along the internal carotid artery in the two groups. The median maximal diameter of the aneurysm was 3.68 ± 0.82 mm in the small aneurysm group and 7.61 ± 1.64 mm in the medium aneurysm group. The neck diameter of the aneurysms was 3.25 ± 0.96 mm in the small aneurysm group and 6.24 ± 1.97 mm in the medium aneurysm group. The distal diameter of the parent artery was 3.55 ± 0.45 mm in the small aneurysm group and 3.56 ± 0.40 mm in the medium aneurysm group. The proximal diameter of the parent artery was 4.21 ± 0.49 mm in the small aneurysm group and 4.47 ± 0.46 mm in the medium aneurysm group (Table 1).

### Implantation/coiling outcome

There was no significant difference in the number (*p* = 1), diameter (*p* = 0.97), or length (*p* = 0.91) of Tubridge in the two groups. Opening failure did not occur during the procedure. The proportion of coil embolization in the medium aneurysm group was higher than that in the small aneurysm group (*p* = 0.08). Branch coverage, such as the ophthalmic artery and posterior communicating artery in the parent artery, occurred in 38 and 18 patients in the small and medium aneurysm groups, respectively. Decreased filling or retention of contrast agents in the aneurysm lumen occurred in 25 (46.30%) and 18 (78.26%) aneurysms in the small and medium aneurysm groups (*p* < 0.05), respectively. There were six patients with new mild cerebral infarction in the small aneurysm group and 0 patients in the medium aneurysm group. No intracranial hemorrhage occurred (Table 2).

**Table 2:** Treatment of Tubridge flow diverter.

**Parameters**	**Small aneurysm**	**Medium aneurysm**	***p*-Value**
No. of stents	39	18	1
**Stent size**			
Diameter	4.03 ± 0.44	4.14 ± 0.52	0.97
Length	29.74 ± 7.60	28.89 ± 5.91	0.91
Unfold failure	0	0	–
Coiling	1 (2.56%)	3 (16.67%)	0.08
Branch coverage	38 (97.44%)	18 (100%)	0.49
Stagnation/decreased contrast filling	25 (46.30%)	18 (78.26%)	< 0.05^*^
Adverse event	6 (15.38%)	0 (0%)	0.08

* Statistically significant.

### Follow-up outcome

The mean times of the last angiographic follow-up were 6.8 ± 1.70 and 8.6 ± 1.30 months in the small and medium aneurysm groups, respectively. A total of three cases were lost to follow-up, including two cases in the small group and one case in the medium group. There were six patients suffering from new mild cerebral infarction with no symptoms after Tubridge flow diversion implantation. The mRS score at the last follow-up was 0.16 ± 0.37 (small aneurysm group) and 0.17 ± 0.57 (medium aneurysm group). Mild stent stenosis occurred in one case of the small aneurysm group, but it did not result in any ischemia symptoms. At the last angiographic follow-up, complete occlusion was achieved in 88.46% of patients in the small aneurysm group and 81.82% of patients in the medium aneurysm group. The mRS at the last follow-up improved in 43.75% (7/16) and 50.00% (4/8) of the two groups, and none worsened in the small and medium aneurysm groups, respectively. There was no morbidity or mortality in any group (Table 3).

**Table 3:** Outcome measures of follow-up.

**Parameters**	**Small aneurysm**	**Medium aneurysm**	***p*-Value**
Cases/aneurysms	37/52	17/22	
Last angiography	6.80 ± 1.70	8.60 ± 1.30	0.65
mRS score	0.16 ± 0.37	0.17 ± 0.57	1
Stent stenosis	1 (2.56%)	0 (0%)	0.49
**Occlusion rate**			
Complete	46 (88.46%)	18 (81.82%)	0.44
Near-complete	6 (11.54%)	4 (18.18%)	
Partial	0 (0%)	0 (0%)	

### The clinical and radiologic outcome of Tubridge flow diversion for tandem aneurysms

There were 19 cases with 39 tandem aneurysms embolized by Tubridge flow diversion, including 18 patients with two tandem aneurysms and one patient with three tandem aneurysms. Decreased filling or retention of contrast agents in the aneurysm lumen occurred in 23 (58.97%) aneurysms of the tandem aneurysms. A total of three patients suffered from new mild cerebral infarction with no symptoms, and no aneurysm ruptured during the operation. The complete occlusion rate of patients with tandem aneurysms on the last angiographic follow-up was 89.75% (35/39; Table 4). The mRS at the last follow-up improved in 50.00% of the cases of the tandem aneurysms group.

**Table 4:** Baseline characteristics, treatment and follow-up of tandem aneurysms.

**Parameters**	**Result**
**Baseline characteristics**	
Patients	
Age (years)	60
Gender (M/F)	7/12
BMI ≥ 24	4 (21.05%)
Aneurysms	
No. (small/medium size)	34 (87.18%)/5 (12.82%)
No. of patients with × aneurysms	
2	18 (94.74%)
3	1 (5.26%)
Side of aneurysms	
Right	22 (56.41%)
Left	17 (43.59%)
Aneurysm locations	
CAVE	5
OPHT	19
p-COMM	2
Aneurysm shape	
Saccular	39
Fusiform	0
Dissecting	0
Aneurysm size (larger/smaller)	
Mean maximal diameter	4.41 ± 1.78/3.07 ± 1.70
Neck size	3.53 ± 1.11/2.57 ± 1.10
Parent artery	
Distal diameter	3.75 ± 0.39
Proximal diameter	4.38 ± 0.45
Length of affected segment	12.24 ± 3.65
Treatment	
No. of stents	19
Stent size	
Diameter	4.13 ± 0.45
Length	31.84 ± 7.65
Unfold failure	0
Coiling	1 (5.26%)
Branch coverage	
OPHT-A	18 (94.74%)
p-COMM-A	1 (5.26%)
Stagnation/decreased contrast filling	23 (58.97%)
Adverse event	3 (15.79%)
Latest follow-up	
Last angiography	7.4 ± 1.89
Stent stenosis	0
Occlusion rate	
Complete	35 (89.75%)
Near-complete	4 (10.25%)
Partial	0

## Discussion

In this article, we report a preliminary experience with Tubridge placement for the treatment of small and medium aneurysms in the anterior circulation. Our results show that the usage of Tubridge for the treatment of small and medium intracranial aneurysms could achieve a high occlusion rate with low morbidity and mortality. In addition, there was no obvious difference in the aneurysm occlusion rate, clinical outcome, or complications between the small and medium groups. These findings mirrored a higher aneurysm occlusion rate and low complication in the two types of aneurysms.

### Flow diversion device for the treatment of small and medium aneurysms

Coiling and stent-assisted coiling are traditional and validated methods for the embolization of small/medium intracranial aneurysms. However, there is still a risk of aneurysm rupture during the insertion of microcatheters and coils into the aneurysm, especially for small aneurysms. Flow diversion devices have rapidly emerged as an essential option for the treatment of intracranial aneurysms, especially for large, giant, and complex aneurysms, due to their high embolization and low complications, which simplifies the procedure by not requiring aneurysmal catheterization (1, 2). However, the use of flow diversion devices in small aneurysms remains to be determined.

Recently, many studies have examined the efficacy and safety of flow diverters for small/medium intracranial aneurysms and indicated high occlusion rates with low morbidity and mortality. The PREMIER study is the first prospective multicenter study to evaluate the use of flow diverters in small/medium, unruptured intracranial aneurysms, which suggests that treatment with the flow-diverting pipeline embolization device is safe and efficacious for small aneurysms, with complication rates comparable with those for traditional endovascular techniques (3). Another real-world study about the safety and efficacy of the pipeline embolization device for small/medium intracranial aneurysms in China also demonstrated high surgical success rates, high occlusion rates, and low morbidity and mortality (4). Therefore, the indication of flow diversion is extended to small and medium aneurysms.

### Flow diversion device for the treatment of tandem aneurysms

Tandem aneurysms are defined as multiple aneurysms located in close proximity to the same parent vessel. The clipping of tandem aneurysms can be challenging, especially when para-ophthalmic or posterior circulation sites are involved. Endovascular treatment of adjacent tandem intracranial aneurysms has been a validated option, including primary coiling, stent-assisted coiling, and flow diversion. However, it will be challenging for embolization adjacent tandem intracranial aneurysms with conventional endovascular techniques, such as primary coiling and stent-assisted coiling, because they usually require repeated catheterization of aneurysms and increase aneurysm rupture during embolization. Flow diversion provides a better reconstruction of the aneurysm neck and has lower recanalization rates, which make it more suitable to treat tandem aneurysms. Especially for small tandem aneurysms, flow diversion implantation with no coiling can achieve a high occlusion rate and low aneurysm rupture rate during the procedure. However, the treatment of tandem aneurysms with flow diversion has rarely been reported in the literature. Lin et al. (5) reported that 13 patients with 28 adjacent tandem aneurysms were treated with pipeline embolization device; complete occlusion was achieved in nine of 10 pipeline embolization device-treated aneurysms. Adeeb and his colleagues indicated that 78 tandem aneurysms underwent 34 pipeline embolization device procedures with high rates of complete occlusion, and symptomatic thromboembolic complications were encountered in 8.8% of procedures (6). A multi-institutional retrospective study released a report that the use of flow diversion for the treatment of tandem cerebral aneurysms had an acceptable safety profile, indicating that it should be considered an effective therapy after reviewing 38 tandem aneurysms of 17 patients (7).

### Safety and efficacy of the Tubridge flow diverter for small/medium aneurysm

The Tubridge is actually a stent-like vessel-reconstruction device designed with a high metal coverage rate and low porosity. It diverts blood flow away from the aneurysm while preserving normal blood flow of the branch artery. It is characterized by a variety of lengths and diameters, radiopaque, flared end, retrievability, and low shortening rate.

As a new endovascular reconstruction tool, the Tubridge flow diverter also shows excellent efficacy and safety for the treatment of intracranial aneurysms, as well as a remarkably equal complete occlusion rate compared with other flow diverters. Currently, many studies have reported that Tubridge is used to treat various complex aneurysms, such as large or giant aneurysms, recurrent aneurysms, vertebral artery dissecting aneurysms, and distal aneurysms, indicating that Tubridge flow diverter can achieve good clinical and radiological outcomes (1, 8–10). However, there was no study reporting the safety and efficacy of Tubridge flow diverter for small/medium and tandem aneurysms.

**Figure 1:**
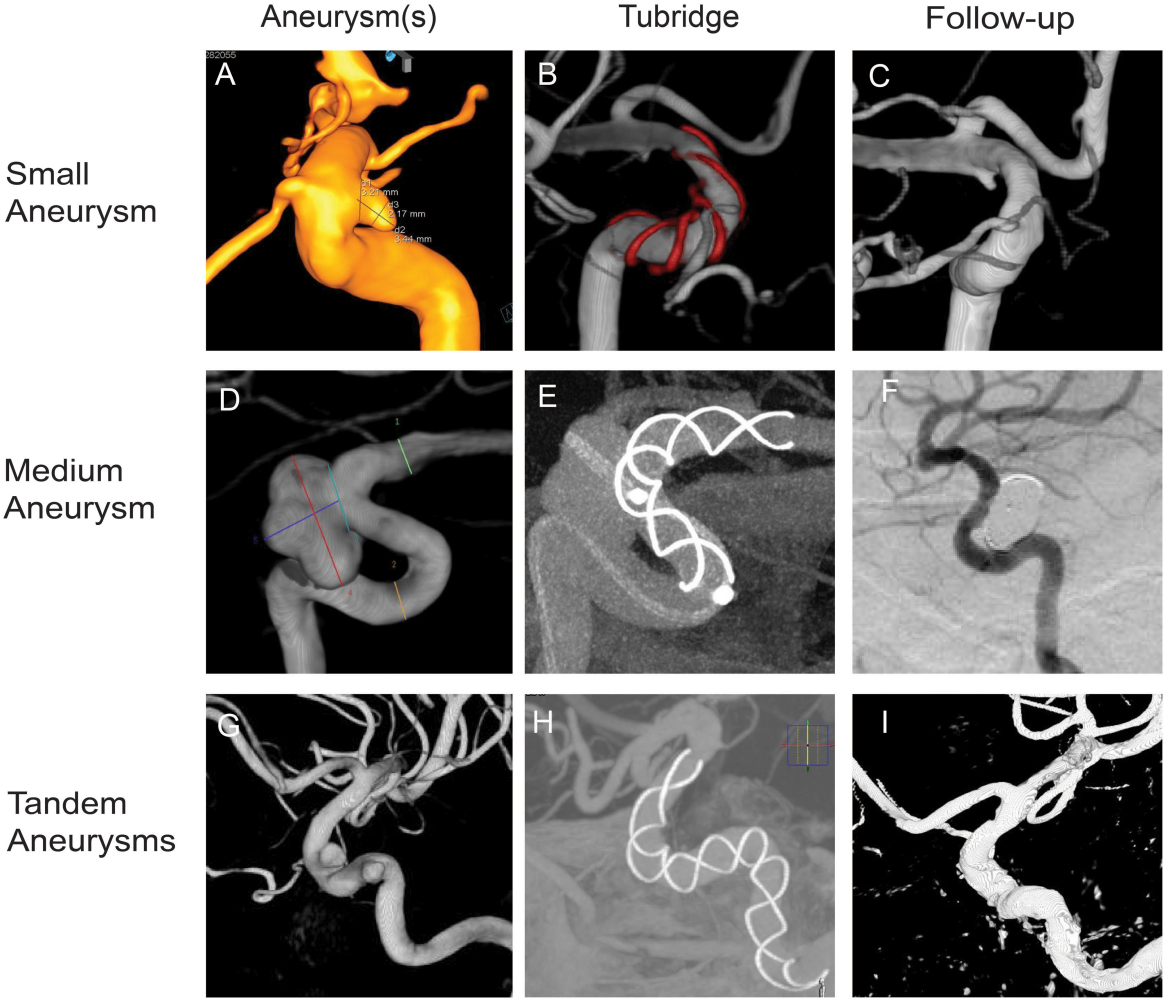
Tubridge flow diverter deployment of small, medium, and tandem aneurysms. **(A)** Small aneurysm, 3.44 mm × 2.17 mm, neck 3.21 mm; parent artery diameter, 4.5–3.3 mm. **(B)** Stent size 4.0–25. **(D)** Medium aneurysm, 10.00 mm × 7.44 mm, neck 5.99 mm; parent artery diameter, 4.2–3.0. **(E)** Stent size, 3.5–25. **(G)** Tandem aneurysms, 4.81 mm × 3.77 mm, neck 4.17 mm. 4.36 mm × 3.09 mm, neck 4.48 mm; parent artery diameter, 5.2–4.5. **(H)** stent size, 5.0–45. **(B, E, H)** Show the opening of the Tubridge flow diverters. **(C, F, I)** Show complete occlusion with the Tubridge flow diverters and coils.

In our study, we evaluate the safety and efficacy of Tubridge in the treatment of small/medium and tandem aneurysms. In total, 57 cases with 77 small/medium aneurysms underwent Tubridge flow diverter implantation (Figure 1), and the complete occlusion rate on the last angiographic follow-up was achieved in 88.46% of the small aneurysms group and 81.82% of the medium aneurysms group. There were six patients with new mild cerebral infarction after the procedure, and all cases had good clinical outcomes at the last follow-up. In addition, there were 19 patients with tandem aneurysms (a total of 39 aneurysms) treated with Tubridge flow diverters, and 89.75% of patients achieved a complete occlusion rate on the last angiographic follow-up, which was comparable with other flow diversion devices.

Compared with other flow diverter devices, Tubridge does not increase the complications, delivery, and opening difficulty of the stent. In contrast, Tubridge has a longer length and a larger diameter choice than other flow diverters, resulting in a lower stenting bridging rate and higher vascular fit. It is reported that minimum distance may affect perioperative complications and complete occlusion for tandem aneurysms (11). Undoubtedly, Tubridge has obvious advantages in the treatment of tandem aneurysms with long minimum distances compared with other flow diversion devices.

### Limitations

We acknowledge that our study is a retrospective study from a single center, and no more patients and time of follow-up were included in our study, although we included parameters about patient characteristics, aneurysms, management, follow-up, imaging studies, and the evaluation of aneurysm occlusion. However, our report on small and medium aneurysms and comparison of the two types of aneurysms treated by Tubridge will make a significant contribution to the existing literature.

## Conclusion

Our preliminary experience demonstrated that the Tubridge flow diverter is a safe and effective stent for the treatment of small and medium aneurysms of the internal carotid artery. Long stents may increase the incidence of cerebral infarction. However, indications and complications require further confirmation, so a multicenter randomized controlled trial with a long-term follow-up is justified and needed.

## Data availability statement

The original contributions presented in the study are included in the article/supplementary material, further inquiries can be directed to the corresponding author.

## Ethics statement

The studies involving human participants were reviewed and approved by the Institutional Review Board of the Hospital. The patients/participants provided their written informed consent to participate in this study.

## Author contributions

WN, SY, CG, YT, and YG contributed to the study conception and design. DX, HY, LZ, and XY conducted the literature review and acquired the data. DX, HY, and LZ performed analysis, interpreted the data, and drafted the manuscript. All authors contributed to the article and approved the submitted version.
